# Osteoglycin as a Potential Biomarker of Mild Kidney Function Impairment in Type 2 Diabetes Patients

**DOI:** 10.3390/jcm10102209

**Published:** 2021-05-20

**Authors:** Sheila González-Salvatierra, Cristina García-Fontana, Francisco Andújar-Vera, Alejandro Borja Grau-Perales, Luis Martínez-Heredia, María Dolores Avilés-Pérez, María Hayón-Ponce, Iván Iglesias-Baena, Blanca Riquelme-Gallego, Manuel Muñoz-Torres, Beatriz García-Fontana

**Affiliations:** 1 Endocrinology and Nutrition Unit, Instituto de Investigación Biosanitaria de Granada (Ibs.GRANADA), University Hospital Clínico San Cecilio, 18016 Granada, Spain; sgsalvatierra@ugr.es (S.G.-S.); franciscoluisandujar@gmail.com (F.A.-V.); mariolaviles@live.com (M.D.A.-P.); mahayon21@gmail.com (M.H.-P.); bgfontana@fibao.es (B.G.-F.); 2 Department of Medicine, University of Granada, 18016 Granada, Spain; luismh95@gmail.com; 3 CIBERFES, Instituto de Salud Carlos III, 28029 Madrid, Spain; 4 Center for Neural Science (CNS), New York University (NYU), New York, NY 10003, USA; agp9975@nyu.edu; 5 Fundación para la Investigación Biosanitaria de Andalucía Oriental (FIBAO), 18012 Granada, Spain; iglesiasbaena@hotmail.com; 6 Department of Preventive Medicine and Public Health, University of Granada, 18016 Granada, Spain; blanca.riquel@gmail.com

**Keywords:** biomarker, diabetic kidney disease, kidney function impairment, osteoglycin, type 2 diabetes

## Abstract

Osteoglycin (OGN) could be a biomarker of mild kidney function impairment in type 2 diabetes (T2D). Our study aimed to determine the association between serum OGN and impaired kidney function risk in T2D patients and to analyze its potential role as an estimator of kidney disturbances in this population. This cross-sectional study included 147 T2D patients (65 ± 8 years, 58.5% males), and 75 healthy controls (63 ± 10 years, 36% males). Circulating OGN levels were determined by ELISA. Linear regression modeling was performed to determine the variables influencing circulating OGN, and an ROC curve was plotted to assess the usefulness of OGN as an estimator of diabetic kidney disease risk. Circulating OGN was significantly increased in T2D patients compared to controls (18.41 (14.45–23.27) ng/mL vs. 8.74 (7.03–12.35) ng/mL; *p* < 0.001). We found a progressive increase in serum OGN according to the severity of kidney impairment in T2D patients (normal kidney function: 16.14 (12.13–20.48) ng/mL; mildly impaired kidney function: 19.15 (15.78–25.90) ng/mL; moderate impaired kidney function: 21.80 (15.06–29.22) ng/mL; *p* = 0.006). Circulating OGN was an independent estimator of mildly impaired kidney function risk in T2D patients. We suggest that serum OGN could act as an albuminuria-independent biomarker of incipient kidney dysfunction in T2D patients.

## 1. Introduction

Osteoglycin (OGN), also known as osteoinductive factor or mimecan, is a secretory protein belonging to class III of the small leucine-rich proteoglycans [1]. OGN is involved in several biological processes [2,3,4] and is related to various pathologies, such as bone fragility, cardiovascular disease (CVD), neurologic disease, ocular diseases, and chronic kidney disease (CKD), among others [2,3]. OGN has a tissue-specific glycosylation site and different post-translational modifications [5] related to its functional role in different locations [2]. OGN participates mainly as a regulator of bone metabolism [6,7], as it is a bone-associated glycoprotein, expressed by osteoblasts [8,9]. Moreover, OGN is a basic component of the vascular extracellular matrix, which is expressed by cardiomyocytes, cardiac fibroblasts, and vascular smooth muscle cells [10,11].

Diabetic kidney disease (DKD) is one of the most frequent complications of type 2 diabetes (T2D). The classic description of DKD involves progressive stages of glomerular hyperfiltration, microalbuminuria, overt proteinuria, and a decline in the estimated glomerular filtration rate (eGFR) [12]. It was widely accepted that patients with DKD develop albuminuria before a decrease in the eGFR. However, these concepts have been increasingly challenged as evidence suggests that DKD is presented in a more heterogeneous manner. Large cross-sectional studies have revealed that a significant proportion of T2D patients with impaired kidney function determined by decreased eGFR values present normal levels of albuminuria [13,14,15,16,17,18]. Therefore, determining biomarkers associated with impaired eGFR could be a useful measurement to analyze the progression to DKD in T2D patients.

Few and contradictory results are known regarding OGN levels in T2D patients with DKD [19,20]. In these studies, OGN was suggested to be a sensitive marker for early microalbuminuria; however, there is no consensus on the level of this protein in T2D patients with DKD compared to healthy subjects. Therefore, the role of OGN in kidney function has not yet been clarified in humans.

Hence, we determine the serum OGN levels in T2D patients with mildly decreased eGFR, in order to assess their significance as a biomarker of impaired kidney function.

## 2. Materials and Methods

### 2.1. Study Population

This cross-sectional study included 222 participants, 147 T2D patients (65 ± 8 years, 58.5% males), and 75 healthy controls (63 ± 10 years, 36% males). T2D was diagnosed according to the American Diabetes Association criteria from 2017 [21]. The recruitment of T2D patients was from 2017 to 2018 in the Endocrinology and Nutrition Unit of the University Hospital Clínico San Cecilio of Granada according to the following criteria: Caucasians having normal values for their blood count, hepatic function, calcium, and phosphorus. The T2D group was classified into two subgroups according to their eGFR: normal eGFR (eGFR ≥ 90 mL/min/1.73 m^2^; mean eGFR 100 ± 7) (*n* = 62) and mildly decreased eGFR (eGFR < 90 mL/min/1.73 m^2^; mean eGFR 69 ± 15) (*n* = 85). Patients with liver, gastrointestinal, and thyroid disease; cancer; dialysis; or renal transplantation were excluded.

Serum samples from healthy controls were supplied by the SSPA Biobank from blood donors of the Andalusian Regional Government Health Service. Healthy donors did not have metabolic diseases as diabetes or infectious, neoplastic, hepatic, cardiovascular, gastrointestinal, central nervous system, or renal diseases (according to section B of Annex II of Royal Decree 1088/2005, of 16 September 2005). All samples used for the study were managed by the SSPA Biobank of the University Hospital Clínico San Cecilio of Granada. Informed consent was obtained from each patient.

This study was conducted with the approval of the Ethics Committee of the University Hospital Clínico San Cecilio of Granada and conformed to the principles of the World Medical Association Declaration of Helsinki (Project ID: 0858-N-17, Research Ethics Committee of Granada Center (CEI-Granada) on 26 April 2017).

### 2.2. Clinical Evaluation

The height, weight, and waist circumference were measured according to standard procedures. The body mass index (BMI) was calculated by the Quetelet formula, weight (kg)/stature (m^2^). The systolic and diastolic blood pressure was measured using a standard electronic sphygmomanometer. Hypertension was defined as values ≥140/90 mmHg and/or antihypertensive treatment. Dyslipidemia was characterized by serum levels of low-density lipoprotein cholesterol (LDL-c) >100 mg/dL, high-density lipoprotein cholesterol (HDL-c) <50 mg/dL, triglycerides (TG) >150 mg/dL, and/or current treatment with lipid-lowering drugs. Patients reported their alcohol use, smoking status, and level of physical activity in response to specific health questionnaires [22].

### 2.3. Biochemical Measurements

Samples of venous blood were taken in the morning after fasting overnight. Serum samples were stored at −80 °C until they were analyzed at the Clinical Analysis Unit of the University Hospital Clínico San Cecilio of Granada. The fasting plasma glucose (FPG), glycated hemoglobin (HbA1c), TG, HDL-c, LDL-c, calcium, and phosphorus were measured using standard automated laboratory techniques. The eGFR was calculated using the Chronic Kidney Disease Epidemiology Collaboration equation [23]. The albumin and creatinine in urine were obtained using a standardized protocol. The abnormal albuminuria was estimated from the urine albumin-to-creatinine ratio (UACR) and was defined as UACR ≥ 30 mg/g.

The calciotropic hormone profile included serum intact parathormone (iPTH) and 25-hydroxyvitamin D (25(OH)D) as determined with the two-site immunoassay (Roche Diagnostics SL, Barcelona, Spain) and the chemiluminescence immune assay (Beckman Coulter UniCel DxI 800, Brea, CA, USA), respectively.

The OGN was determined in duplicate by the enzyme-linked immunosorbent assay (ELISA) method developed by Cloud-Clone Corp. (Houston, TX, USA), following the manufacturer’s protocol. Precision testing was performed by the determination of the intra-assay and inter-assay coefficients of variation of OGN (<10% and <12%, respectively).

Fibroblast growth factor 23 (FGF-23) was measured in duplicate using ELISA (Biomedica). The intra- and inter-assay coefficients of variation of FGF-23 were <6% and <8%, respectively.

### 2.4. Statistical Analysis

Analyses were performed using SPSS version 22.0 software (SPSS, Inc., Chicago, IL, USA). The data were expressed as the means ± standard deviation (SD) for the normally distributed variables and as the median with the interquartile range (IQR) for variables that were not normally distributed. The data for categorical variables are presented as percentages. A Shapiro–Wilk test was used to test the normality of the distribution of the continuous variables. The mean values between groups were compared using the unpaired Student’s *t*-test for continuous and normally distributed variables. The Mann–Whitney U test and Kruskal–Wallis test were used to compare the variables that were not normally distributed. When the comparison between groups required an adjustment by covariates, an analysis of covariance (ANCOVA) was performed. The χ^2^ test was used to compare categorical variables between groups.

Associations between continuous variables were described by Spearman’s correlation coefficients. Multiple linear regression modeling was performed to determine the variables independently associated with the OGN (dependent variable), including the quantitative and qualitative variables linked in the bivariate analysis and other variables biologically associated with OGN as independent variables. 

To identify OGN as an independent predictor of impaired kidney function, multiple logistic regression modeling was performed, including mildly decreased eGFR as a dependent variable. The independent variables included in the model were the established factors related to impaired kidney function risk in addition to the OGN level. The usefulness of serum OGN as an estimator of impaired kidney function risk was assessed using a receiver operating characteristic (ROC) curve. The area under the curve (AUC) indicates the probability of predicting an event. AUC values greater than 0.75 indicate good predictive performance.

The statistical significance was set at *p* < 0.05 (two-tailed) and *p* < 0.10 for multiple linear regression analysis.

## 3. Results

### 3.1. Characteristics of the Study Population

The clinical, anthropometric, and biochemical parameters of T2D participants according to normal or mildly impaired kidney function are summarized in Table 1.

The recruited groups were homogenous, and there were no differences between them in sex, weight, height, BMI, waist circumference, or diabetes duration. Regarding age, there was a significant difference between groups. Most of the clinical parameters were also comparable between groups except for hypertension, dyslipidemia, and CVD. The biochemical parameters differed between the groups in terms of the serum levels of iPTH, FGF-23, and OGN.

### 3.2. Influence of Diabetes Status, Sex, and eGFR on the Serum OGN Levels

**Figure 1:**
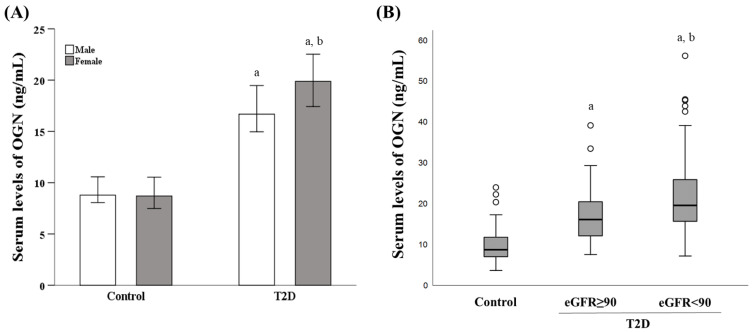
Serum OGN levels in control subjects and T2D patients according to sex and eGFR. (**A**) Serum levels of OGN (median ± 95% confidence interval (CI)) in the control and T2D groups for both sexes. The Kruskal–Wallis test was used for comparisons between groups. a = *p <* 0.05 vs. control of the same sex; b = *p <* 0.05 vs. male in T2D patients. (**B**) Box plot of serum OGN levels in controls, T2D patients with normal eGFR (eGFR ≥ 90 mL/min/1.73 m^2^), and T2D patients with mildly decreased eGFR (eGFR < 90 mL/min/1.73 m^2^). Box plot represents the minimum value, 25th percentile, median, 75th percentile, maximum value, and outliers for each group. The Kruskal–Wallis test was used for comparisons between groups. a = *p <* 0.05 vs. control; b = *p <* 0.05 vs. T2D with eGFR ≥ 90 mL/min/1.73 m^2^. OGN: osteoglycin; T2D: type 2 diabetes; eGFR: estimated glomerular filtration rate.

The serum OGN levels were significantly higher in T2D patients (*n* = 136, 57% males) than in control subjects (*n* = 75, 36% males) (18.41 (14.45–23.27) ng/mL vs. 8.74 (7.03–12.35) ng/mL; *p* < 0.001). When T2D patients and control subjects were further divided according to sex, the significant differences in OGN levels remained for both males (16.68 (13.44–20.77) ng/mL vs. 8.79 (7.64–11.17) ng/mL; *p* < 0.001), and females (19.88 (15.70–26.30) ng/mL vs. 8.70 (6.65–12.41) ng/mL; *p* < 0.001). We found that the OGN levels were significantly higher in females than in males in the T2D group (19.88 (15.70–26.30) ng/mL vs. 16.68 (13.44–20.77) ng/mL; *p* = 0.009), but no differences were found for the healthy controls according to sex (Figure 1A).

The comparison of serum OGN levels between control subjects and T2D patients with normal (*n* = 58, 55% males) and mildly decreased (*n* = 78, 59% males) eGFR revealed significant differences between all groups (*p* < 0.001). The control group showed lower levels of serum OGN (8.74 (7.03–12.35) ng/mL). T2D patients with normal eGFR showed lower circulating OGN levels compared to T2D patients with mildly decreased eGFR (16.14 (12.13–20.48) ng/mL vs. 19.59 (15.70–25.90) ng/mL; *p* = 0.013) (Figure 1B). After adjusting by age and sex, this trend in OGN levels remained unchanged (18.02 ± 1.09 vs. 21.59 ± 0.93; *p* = 0.017).

In the group of T2D patients with mildly decreased eGFR, there is a 20.5% of T2D patients with a moderate decrease in eGFR (<60 mL/min/1.73 m^2^). In this subgroup (*n* = 16, 65 ± 8 years, 60% males), the highest levels of OGN were observed (21.80 (15.06–29.22) ng/mL) with significative differences compared to normal eGFR group (*p* = 0.022).

### 3.3. Determinants of Serum OGN Levels in the T2D Group

**Figure 2:**
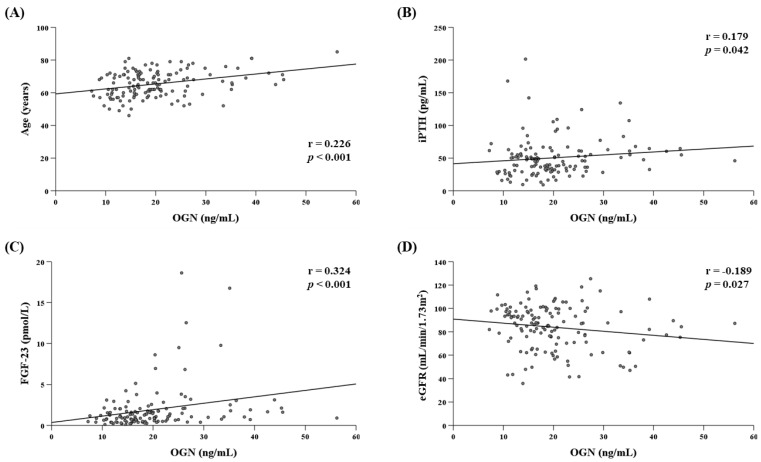
Scatter plots showing the correlation (Spearman’s test) between OGN (ng/mL) and: age (years): (**A**), iPTH (pg/mL), (**B**), FGF-23 (pmol/L), (**C**), and eGFR (mL/min/1.73 m^2^), (**D**), in T2D patients. OGN: osteoglycin; iPTH: intact parathormone; FGF-23: fibroblast growth factor 23; eGFR: estimated glomerular filtration rate; T2D: type 2 diabetes.

We found a positive correlation between the serum level of OGN with age (r = 0.226; *p* < 0.001), iPTH (r = 0.179; *p =* 0.042), and FGF-23 (r = 0.324; *p* < 0.001) and a negative correlation with eGFR (r = −0.189; *p =* 0.027) in T2D patients (Figure 2).

To analyze the variables that influence the level of OGN, a model of multiple linear regression analysis was performed including the variables associated with OGN in the previously performed bivariate analysis (age, sex, iPTH, FGF-23, and eGFR) in addition to the diabetes duration, insulin treatment, UACR, presence of osteoporosis, and presence of CVD as independent variables. The results showed that the variables independently associated with the OGN serum level were age and FGF-23, as shown in Table 2.

The presence of CVD was not independently associated with serum OGN level, although it bordered on significance (*p* < 0.061). The comparison of serum OGN levels in T2D patients according to the prevalence of CVD after age and sex adjustment revealed significant differences between both groups (*p* = 0.041). T2D patients with CVD (*n* = 45, 67 ± 7 years, 79% males) showed lower circulating OGN levels compared to T2D patients without CVD (*n* = 91, 65 ± 8 years, 47% males): (17.88 (15.39–20.38) ng/mL vs. 21.15 (19.44–22.86) ng/mL).

### 3.4. Association between FGF-23 Levels with Kidney Function and CVD

The serum FGF-23 levels were higher in T2D patients (*n* = 132, 57% males) compared to that in controls subjects (*n* = 75, 36% males) although not significantly (1.16 (0.63–2.05) pmol/L vs. 1.02 (0.47–1.85) pmol/L; *p* = 0.186). When T2D patients were divided according to eGFR values, we observed higher circulating FGF-23 levels in the group with mildly decreased eGFR (*n* = 76, 59% males) compared to those with normal values (*n* = 56, 54% males) (*p* = 0.028) (Table 1). When patients with prevalent CVD were excluded from the analysis, we similarly observed higher FGF-23 levels in patients with decreased eGFR (*n* = 46) compared to those with normal eGFR values (*n* = 43): (1.55 (0.93–2.36) pmol/L vs. 0.84 (0.44–1.90) pmol/L; *p* = 0.005). No significative differences were found between T2D patients according to the prevalence of CVD independently of the kidney function.

### 3.5. Usefulness of the OGN Serum Level to Estimate Impaired Kidney Function Risk in T2D Patients

Logistic regression modeling was performed to assess the variables related to impaired kidney function risk in T2D patients. The independent variables included in the model were age, hypertension, dyslipidemia, HbA1c levels (categorized according to the cutoff point of 7%), tobacco use, years of diabetes duration, presence of CVD, presence of osteoporosis, and UACR in addition to the OGN serum level. We found that, in addition to age (OR = 1.08; 95% CI (1.01/1.15); *p* = 0.021), the serum OGN level was an independent estimator of impaired kidney function risk (OR = 1.07; 95% CI (1.01/1.14); *p* = 0.029) in T2D patients. 

**Figure 3:**
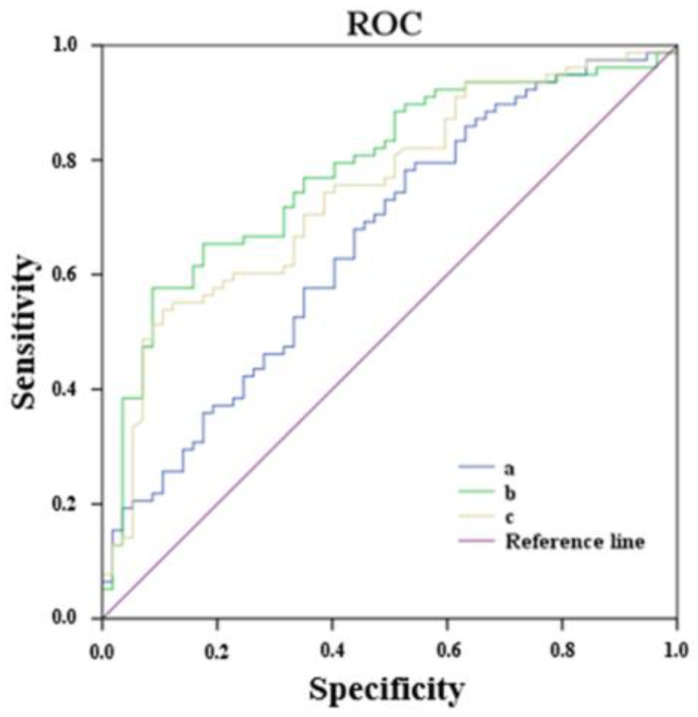
ROC curve for the usefulness of serum OGN level as an estimator of impaired kidney function in T2D patients. (a) OGN serum levels; AUC = 0.658; *p* = 0.002. (b) Age, hypertension, dyslipidemia, HbA1c levels (categorized according to the cutoff point of 7%), tobacco use, years of diabetes duration, and OGN serum levels; AUC = 0.782; *p* < 0.001. (c) Age, hypertension, dyslipidemia, HbA1c levels (categorized according to the cutoff point of 7%), tobacco use, and years of diabetes duration; AUC = 0.748; *p* < 0.001. ROC: receiver operating curve; OGN: osteoglycin; T2D: type 2 diabetes; AUC: area under the curve; HbA1c: glycated hemoglobin.

ROC curve analysis was performed to assess the usefulness of the serum level of OGN as an estimator of impaired kidney function risk. Two different models were assessed, including the main impaired kidney function risk factors (age, hypertension, dyslipidemia, HbA1c level, tobacco use, and years of diabetes duration) with and without the serum OGN level (Figure 3).

The AUC of the model without OGN was 0.748, whereas the AUC of the model including OGN was 0.782 (*p* < 0.001 for both).

## 4. Discussion

Our cross-sectional study shows higher OGN levels in T2D patients compared to healthy controls for the first time. T2D patients with normal eGFR show lower circulating OGN levels than T2D patients with mild or moderate impaired eGFR, independent of sex and age. The serum OGN levels are independently associated with mildly impaired kidney function in T2D patients. This suggests that circulating OGN may be a biomarker of incipient impairment of kidney function, independently of the presence of albuminuria in T2D patients.

Little is known regarding the serum levels of OGN in T2D and control subjects with contradictory results. The increased levels of serum OGN observed in T2D patients compared to healthy controls, especially in those with decreased eGFR values agree with the findings reported by Wang et al., who found increased concentrations of OGN in T2D patients with diabetic nephropathy compared to T2D patients without diabetic nephropathy and healthy controls [19]. In contrast, Wei et al. reported that the decline of serum OGN levels was closely related to the development and pathogenesis of diabetic nephropathy suggesting that low serum OGN levels could act as an independent diagnostic marker of diabetic nephropathy associated with microalbuminuria in T2D patients [20]. Similarly, a study in mice with CKD showed that decreased serum OGN level was positively associated with impaired kidney function [24]. The aforementioned studies associate the OGN serum levels with kidney status independently of the presence of T2D. However, our study is the first to show higher circulating OGN levels in T2D patients regardless of kidney function.

The higher levels of FGF-23 in T2D patients compared to control subjects could partly explain the elevation of serum OGN in this group. Consistently, studies reported increased FGF-23 serum levels in the T2D population [25,26]. The presence of T2D implies a progressive deterioration of kidney function, which is associated with increased serum levels of FGF-23 and iPTH as we observed in the T2D subjects with an incipient decrease in glomerular filtration. The elevation of iPTH in these patients could be explained by its positive correlation with FGF-23 as has been reported in CKD patients [27,28]. However, a review addressing the role of FGF-23 in clinical outcomes in T2D patients has shown that the mechanism by which an increase in FGF-23 occurs in T2D patients is unclear [29]. Most studies suggest that the increase in FGF-23 may be associated with the presence of CVD and may act as a predictor of cardiovascular mortality in the T2D population [30,31]. Considering this, the increased FGF-23 levels observed in the T2D group of patients with decreased eGFR levels could be due to the higher prevalence of CVD in this group. However, our results suggest that the increased FGF-23 is associated more with kidney function disturbances rather than with CVD in our study population.

Most of the studies have shown a strong and independent association between FGF-23 concentrations and greater risk of end-stage renal disease in advanced-stage CKD patients [32,33], thereby making it an independent risk factor for mortality in this population [32,34]. However, a long-term prospective study including patients with nondiabetic CKD has identified FGF-23 as a novel risk marker for the progression of CKD in mild to moderate CKD patients [27].

Regarding the T2D population, there is some controversy. Several studies have pointed to serum levels of FGF-23 as predictors for renal outcomes and progression to end-stage renal disease in T2D patients [35,36]. It has been revealing that FGF-23 is a novel independent predictor of the progression of renal disease in patients with macroalbuminuric diabetic nephropathy [27]. However, a recent multicenter study, reported a lack of association between FGF-23 and early kidney decline in T2D patients [37]. Based on our findings, we suggest that the ability of FGF-23 to predict early stages of renal impairment could be limited [30,31]. We consider that it could be due to its relationship with albuminuria since most studies associate elevated FGF-23 levels with nephropathy including albuminuria. In our study population, most of the T2D patients with mildly decreased eGFR had normoalbuminuric (75.9%). Our results showed that serum OGN levels are related to eGFR values, independently of the albuminuria. In this context, we suggest that, as OGN is an albuminuria-independent biomarker, it could be a better predictor than FGF-23 in T2D patients with early kidney impairment (data not shown).

Although there are markers associated with kidney impairment, the predictions for the progression of DKD in T2D patients based on clinical parameters are currently poor. The majority of the criteria for early kidney function deterioration considers albuminuria in combination with eGFR as the main clinical factors despite its modest predictive ability [25]. However, a decrease in eGFR may occur as an initial sign of kidney impairment without the presence of albuminuria as reported by Penno et al., who showed an association between decreased eGFR and the risk of death irrespective of albuminuria in T2D patients [15]. Accordingly, research reported that the majority of T2D patients with reduced eGFR had normoalbuminuric [16] and a significant risk for coronary artery disease in T2D patients with reduced eGFR independently of albuminuria [26].

Considering this, there is a need to search for effective intervention strategies and public health policies focused on the detection of incipient impaired kidney function in T2D patients, due to the rising mortality rate associated with this non-albuminuria DKD phenotype [15]. In this context, much of the research conducted in the last decade has endeavored to identify biomarkers for DKD progression. Some proposed useful biomarkers to measure the progression of advanced kidney damage [35,36]. However, few studies have determined biomarkers that can predict the eGFR decline in T2D patients when combined with commonly available clinical risk factors. Heinzel et al. identified some candidate biomarkers associated with eGFR in a longitudinal study conducted in T2D patients with the eGFR maintained at baseline. However, their predictive power was low [37]. Our results showed that the OGN serum level was an independent estimator of impaired kidney function risk in T2D patients by increasing the risk by 8% for a 1 ng/mL increase in the serum OGN regardless of other comorbidities such as CVD or osteoporosis. Our ROC curve analysis revealed that the inclusion of serum OGN levels, in addition to age and impaired kidney function–related variables improved the prediction model for mildly impaired kidney function in T2D patients.

The positive correlation observed between serum OGN levels and age may be due to their close relationship with age-related renal function loss in T2D patients [38,39]. Although the T2D patients with eGFR values below 90 mL/min/1.73 m^2^ were significantly older than those with normal eGFR values, the differences between groups remained significant after adjustment for age and sex. Our results showed that in addition to age, the serum FGF-23 levels could influence serum OGN. Based on these findings, the age and the higher serum levels of iPTH, and in particular, of FGF-23, could explain the increased OGN in T2D patients with eGFR values below 90 mL/min/1.73 m^2^.

On the other hand, the fact that prevalent CVD is so close to significance as a predictor variable of OGN as shown in our results suggests that there may be a relationship between OGN levels and CVD. The role of OGN in CVD is controversial to date. Some studies’ results have reported a close relationship between OGN levels and the risk of suffering CVD although the mechanism of action of OGN is unclear. In this line, a couple of studies found an association between high serum OGN levels and poor coronary collateralization in patients with coronary artery disease [40,41], as well as increased arterial stiffness in hypertensive patients [42]. Cheng et al. pointed out that OGN could be used as a prognostic biomarker in patients with coronary artery disease, and it proved to be a predictor for the incidence of cardiovascular events and mortality within this population [11]. Although most of the studies postulate an association between higher OGN levels and vascular damage, Van Aelst et al. reported that the increased OGN expression is essential in the infarct scar promoting proper collagen maturation and protecting against cardiac disruption in humans [4]. In contrast, another study found no association between the circulating levels of OGN and the progression of atherosclerosis [43]. We found lower serum circulating OGN levels in T2D patients with CVD than in T2D patients without CVD. Therefore, our results suggest that the increased serum OGN levels observed in T2D patients with mildly impaired kidney function (eGFR < 90 mL/min/1.73 m^2^) are mainly related with renal rather than vascular damage. Endorsing our findings, a recent study observed increased immunostaining of OGN in human atherosclerotic carotid plaques from patients with lower eGFR values [44]. However, further studies are needed to clarify the relationship between OGN and CVD.

Although future longitudinal studies are needed, our preliminary results place OGN as a promising biomarker that deserves future research to confirm its potential role as an early predictor of kidney damage in daily clinical practice.

Our cross-sectional study has some limitations. First, the cross-sectional design precludes any determination of causality in our findings. Thus, we cannot assure whether FGF-23 influences the increase in OGN levels or the other way around. Second, we do not have many biochemical determinations in the control group, which could provide valuable information for the comparative study of OGN between T2D and control subjects. Finally, our study was conducted in a specific population of Caucasian T2D and healthy subjects, which prevents guaranteeing the same results in other ethnic or study groups. The strengths of this study lie in the evaluation of circulating OGN in T2D patients with an exhaustive evaluation of the biochemical and clinical parameters. In addition, we considered potential confounders, such as age, sex, diabetes duration, and current medications.

## 5. Conclusions

The main findings of this study suggest that the elevation of OGN related to mildly impaired kidney function could involve a specific role of OGN in this process acting as an albuminuria-independent biomarker of incipient impaired kidney function in T2D patients. Future longitudinal studies are required to understand the mechanisms through which the upregulation of OGN influences the risk of impaired kidney function in T2D patients and to confirm the potential usefulness of serum OGN as a potential biomarker of kidney status in clinical settings to establish preventive and therapeutic approaches in the T2D population.

## Figures and Tables

**Table 1:** Comparison between eGFR ≥ 90 mL/min/1.73 m^2^ and eGFR < 90 mL/min/1.73 m^2^ in T2D patients.

	eGFR (mL/min/1.73 m^2^)		
	eGFR ≥ 90	eGFR < 90	*p*
Patients *(n)*	62	85	
Men/women (%)	56/44	60/40	0.666
Age (years)	62 ± 7	68 ± 8	<0.001 *
eGFR (mL/min/1.73 m^2^)	100 ± 7	69 ± 15	<0.001 *
CLINICAL EVALUATION			
Body weight (kg)	86 ± 15	87 ± 13	0.838
Height (cm)	165 ± 0.09	165 ± 0.08	0.717
BMI (kg/m^2^)	32 ± 5	32 ± 4	0.944
Waist circumference (cm)	106 ± 11	106 ± 10	0.900
Diabetes duration (years)	14 ± 10	15 ± 9	0.468
Systolic blood pressure (mmHg)	133 ± 17	137 ± 18	0.133
Diastolic blood pressure (mmHg)	79 ± 9	79 ± 12	0.833
UACR ≥ 30 mg/g (%)	21	24	0.657
Hypertension (%)	76	92	0.007 *
Dyslipidemia (%)	82	93	0.045 *
CVD (%)	25.8	43.5	0.027 *
Osteoporosis (%)	9.7	5.9	0.399
Smoker or ex-smoker (%)	48	46	0.843
Alcohol consumption excessive (%)	20	13	0.271
Sedentarism (%)	15	17	0.735
CURRENT MEDICATION USE			
Insulin (%)	10	14	0.756
Oral antidiabetic drugs (%)	31	28	0.551
Insulin + Oral antidiabetic drugs (%)	59	58	0.423
BIOCHEMICAL MEASUREMENTS			
FPG (mg/dL)	150 ± 52	150 ± 55	0.989
HbA1c (mmol/mol)	62 ± 16	63 ± 15	0.792
HbA1c (%)	7.8 ± 1.4	7.9 ± 1.3	0.792
TG (mg/dL)	158 ± 71	166 ± 85	0.568
HDL-c (mg/dL)	47 ± 13	44 ± 10	0.128
LDL-c (mg/dL)	98 ± 44	88 ± 37	0.127
Calcium (mg/dL)	9.8 ± 0.4	9.7 ± 0.4	0.368
Phosphorous (mg/dL)	3.4 ± 0.5	3.3 ± 0.4	0.098
25(OH)D (ng/mL)	20 ± 8	22 ± 9	0.167
iPTH (pg/mL)	44 ± 25	56 ± 34	0.029 *
FGF-23 (pmol/L)	0.86 (0.47–1.70)	1.25 (0.77–2.44)	0.028 *
OGN (ng/mL)	16.14 (12.13–20.48)	19.59 (15.70–26.90)	0.002 *

T2D: type 2 diabetes; eGFR: estimated glomerular filtration rate; BMI: body mass index; UACR: urine albumin-to-creatinine ratio; CVD: cardiovascular disease; FPG: fasting plasma glucose; HbA1c: glycated hemoglobin; TG: triglycerides; HDL-c: high-density lipoprotein cholesterol; LDL-c: low-density lipoprotein cholesterol; 25(OH)D: 25-hydroxyvitamin D; iPTH: intact parathormone; FGF-23: fibroblast growth factor 23; OGN: osteoglycin. The data for continuous and normally distributed variables are presented as the mean ± SD. The data for continuous variables that are not normally distributed are presented as the median followed by the interquartile range in brackets. The data for categorical variables are presented as percentages. Student’s *t*-test and the Mann–Whitney *U* test were used for comparisons of continuous and normally or not normally distributed variables, respectively, between groups. The χ^2^ test was used for the comparison of categorical variables between groups. The * symbol represents statistically significant differences (*p* < 0.05) between groups.

**Table 2:** Multiple linear regression analysis of variables independently associated with serum OGN levels in T2D patients.

Variables	B	95% CI (Lower Limit/Upper Limit)	*p*
Age	0.319	0.091/0.547	0.007 *
Sex	−3.090	−6.550/0.371	0.080
iPTH	0.018	−0.043/0.080	0.554
FGF-23	0.838	0.275/1.400	0.004 *
eGFR	−0.036	−0.127/0.056	0.440
Insulin treatment	−0.089	−0.274/0.097	0.347
Current medication	−3.131	−6.796/0.535	0.093
UACR ≥ 30 mg/g	0.024	−0.003/0.051	0.076
Presence of osteoporosis	−0.559	−6.128/5.010	0.842
Presence of CVD	−3.243	−6.641/0.156	0.061

OGN: osteoglycin; T2D: type 2 diabetes; CI: confidence interval; iPTH: intact parathormone; FGF-23: fibroblast growth factor 23; eGFR: estimated glomerular filtration rate; UACR: urine albumin-to-creatinine ratio; CVD: cardiovascular disease. The * symbol represents statistically significant differences (*p* < 0.05).

## Data Availability

The data sets generated and/or analyzed during the current study are available from the corresponding author on reasonable request.
